# Revolutionizing Health Care: The Transformative Impact of Large Language Models in Medicine

**DOI:** 10.2196/59069

**Published:** 2025-01-07

**Authors:** Kuo Zhang, Xiangbin Meng, Xiangyu Yan, Jiaming Ji, Jingqian Liu, Hua Xu, Heng Zhang, Da Liu, Jingjia Wang, Xuliang Wang, Jun Gao, Yuan-geng-shuo Wang, Chunli Shao, Wenyao Wang, Jiarong Li, Ming-Qi Zheng, Yaodong Yang, Yi-Da Tang

**Affiliations:** 1 Department of Cardiology State Key Laboratory of Cardiovascular Disease Fuwai Hospital, National Center for Cardiovascular Diseases, Chinese Academy of Medical Sciences and Peking Union Medical College Beijing China; 2 Pengcheng Laboratory Shenzhen, Guangdong China; 3 School of Disaster and Emergency Medicine Tianjin University Tianjin China; 4 Institute for Artificial Intelligence Peking University Beijing China; 5 China Telecom Beijing China; 6 Division of Emerging Interdisciplinary Areas Hong Kong University of Science and Technology Hong Kong China (Hong Kong); 7 Institute for Artificial Intelligence Hefei University of Technology Hefei, Anhui China; 8 Department of Cardiology the First Hospital of Hebei Medical University Graduate School of Hebei Medical University Shijiazhuang, Hebei China; 9 Department of Cardiology and Institute of Vascular Medicine Key Laboratory of Molecular Cardiovascular Science, Ministry of Education Peking University Third Hospital Beijing China; 10 Henley Business School University of Reading RG6 6UD United Kingdom

**Keywords:** large language models, LLMs, digital health, medical diagnosis, treatment, multimodal data integration, technological fairness, artificial intelligence, AI, natural language processing, NLP

## Abstract

Large language models (LLMs) are rapidly advancing medical artificial intelligence, offering revolutionary changes in health care. These models excel in natural language processing (NLP), enhancing clinical support, diagnosis, treatment, and medical research. Breakthroughs, like GPT-4 and BERT (Bidirectional Encoder Representations from Transformer), demonstrate LLMs’ evolution through improved computing power and data. However, their high hardware requirements are being addressed through technological advancements. LLMs are unique in processing multimodal data, thereby improving emergency, elder care, and digital medical procedures. Challenges include ensuring their empirical reliability, addressing ethical and societal implications, especially data privacy, and mitigating biases while maintaining privacy and accountability. The paper emphasizes the need for human-centric, bias-free LLMs for personalized medicine and advocates for equitable development and access. LLMs hold promise for transformative impacts in health care.

## Introduction

Recent advancements in artificial intelligence (AI) have catalyzed the development and significant breakthroughs of large language models (LLMs), placing them at the forefront of AI research [1-4]. LLMs are deep learning models that generate human-like text by predicting the next word in a sequence based on statistical patterns learned from vast text data. These models leverage deep learning algorithms to interpret and generate natural language, using extensive corpus data to enhance pretrained language models, a cornerstone of natural language processing (NLP) [5,6]. Characterized by their immense scale, these models often consist of hundreds of millions to billions of parameters and are trained on vast textual datasets [7,8]. Their ability to efficiently process natural language data with minimal human intervention, capturing intricate grammatical structures, lexical nuances, and semantic contexts, is noteworthy. Globally recognized LLMs include the ChatGPT series, BERT (Bidirectional Encoder Representations from Transformer), PaLM, LaMDA, and Meta’s Llama series, with China contributing models such as Baidu’s “Wenxin Yiyan,” 360’s LLM, Alibaba’s “Tongyi Qianwen,” and SenseTime’s LLM [9]. The evolution of LLMs represents over 7 years of relentless technological innovation and research, marking a significant milestone in AI development since the inception of the Turing machine.

LLMs primarily function to comprehend, generate, and interact through language. In NLP tasks, such as text classification, named entity recognition, and sentiment analysis, their proficiency is unparalleled [10-12]. Beyond these applications, LLMs are expanding their influence. In mathematics, they assist in solving complex problems and contributing to mathematical proofs [13]. In software development, their capabilities include automatic code generation, debugging assistance, and complex algorithm explanation [14]. Intriguingly, LLMs are venturing into artistic creation, exhibiting talent in generating poetry, stories, and music [15,16].

**Figure 1:**
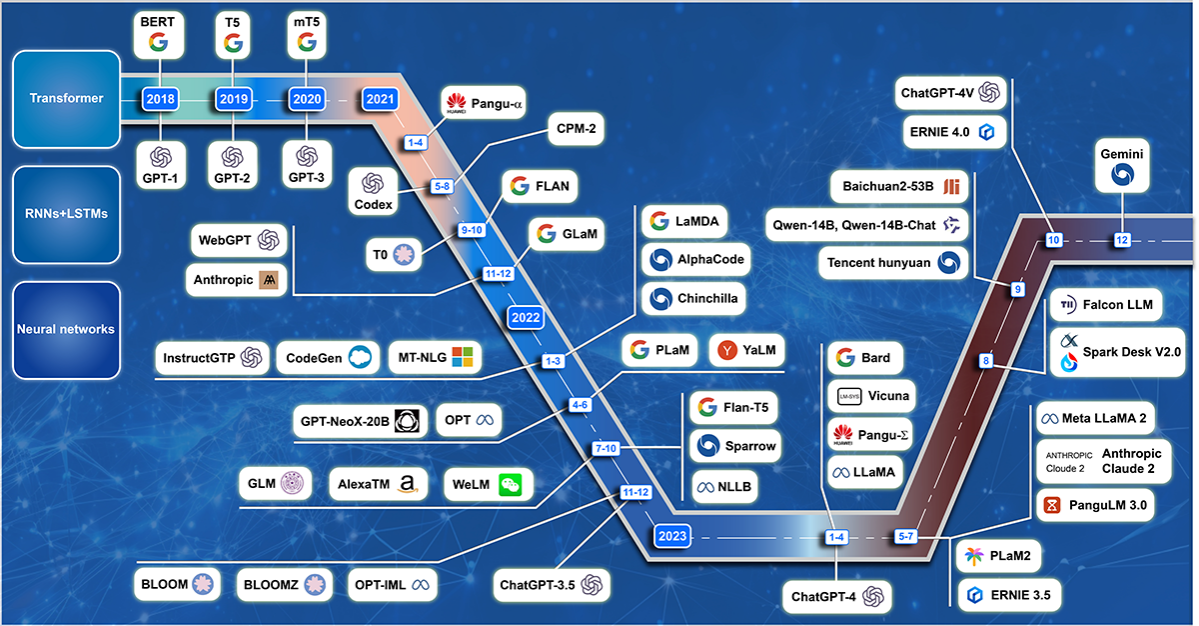
Timeline of mainstream LLMs commercially available to the public. The technological evolution of LLMs, highlighting several key technologies and models. It includes RNNs and LSTMs from the 1990s, Google’s Transformer model introduced in 2017, Google’s BERT model released in 2018, and the GPT series by OpenAI. Specific emphasis is placed on three major milestones: the first open-source LLM—GPT-2, and the first widely acclaimed LLM—GPT-3. These developments signify major advancements in LLMs within the field of natural language processing. BERT: Bidirectional Encoder Representations from Transformers; LLM: large language model; LSTM: long short-term memory network; RNN: recurrent neural network.

In the medical domain, LLMs are poised to revolutionize clinical decision support. They can assist health care professionals in diagnosing diseases with enhanced accuracy and speed, provide treatment recommendations, and facilitate the analysis of medical records by processing large volumes of medical data [17-20]. They are instrumental in swiftly navigating vast medical literature, providing health care professionals with essential research, guidelines, and information, thus saving time and grounding medical treatments in current knowledge [21-25]. Additionally, LLMs can interact directly with patients, offer medical consultations, and handle document processing efficiently [26-28]. For example, health care professionals use LLMs to assist in diagnosing diseases by quickly processing and interpreting large volumes of patient data such as electronic health records and imaging results. Clinicians also leverage LLMs for treatment planning, where the models suggest potential treatment options based on the latest medical guidelines and patient-specific data. Moreover, LLMs are used in streamlining administrative tasks, such as generating and managing medical documentation, allowing clinicians to spend more time with their patients. Their role in drug research and development is also emerging, aiding in new drug discoveries through detailed analysis of chemical and biological data [29,30]. As such, LLMs are reshaping research methodologies and applications across various fields, particularly in medicine, equipping doctors with advanced tools for more accurate and efficient diagnosis and treatment, while offering patients more convenient and effective medical services. The potential for broader applications of LLMs in the medical field is vast, and there is a strong rationale to expect their significant impact on future health care advancements (Figure 1).

## LLM Technical Background and Hardware Infrastructure

The evolution of LLMs, like OpenAI’s GPT-3 and Google’s BERT, has been monumental, driven by advancements in AI chip computing power and large, high-quality datasets [31]. The Transformer model, introduced by Google in 2016, underpins this progress, predicting words in sentences based on statistical correlations [32,33]. Notably, GPT-3 in 2020 showcased the significance of model size and data quality.

The operation and training of LLMs, such as ChatGPT, require substantial hardware infrastructure [34]. This includes graphics processing units (GPUs) or tensor processing units (TPUs) with thousands of cores, extensive RAM (several terabytes), over 48 GB of VRAM on GPUs, high-performance solid-state drives, and fast, low-latency networks (10 to 100 Gbps) [35,36]. Effective cooling systems and reliable power supplies are also essential. Compatibility with software frameworks, like TensorFlow and PyTorch, is necessary for optimizing training and deployment. The training of GPT-3, for instance, costs around US $1.4 million, and operational costs for models, like ChatGPT, can reach up to US $700,000 daily, with significant energy consumption.

Future technology advancements are expected to reduce the costs and improve the efficiency of LLMs. Progress in GPU and TPU technologies, along with hardware tailored for LLM training, will drive efficiency. Compact model structures through knowledge distillation, model pruning, transfer learning, energy-efficient practices, distributed training, and edge computing are anticipated. Semisupervised and self-supervised learning methods will also play a role in training models with fewer labeled datasets [37,38]. ChatGPT’s recent updates showcase improvements in response speed, handling complex queries, multimodal functionality, global language support, and enhanced privacy and security measures [39].

**Figure 2:**
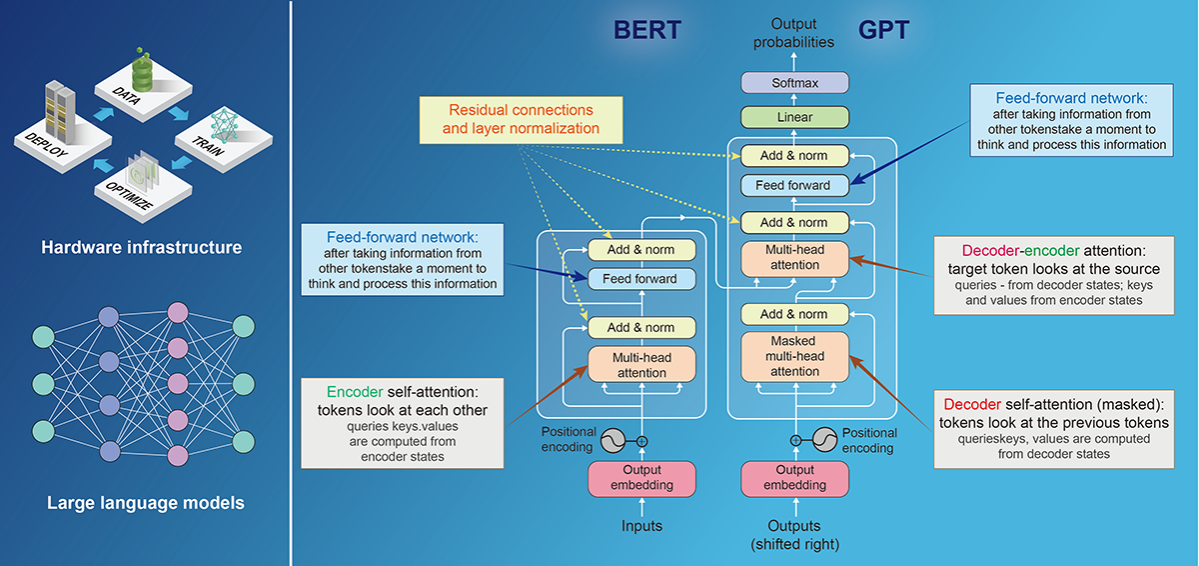
The architectural designs of LLMs: a study of self-attention mechanisms and structural variations. The image depicts the hardware infrastructure for LLMs and their implementation in the BERT and GPT models. On the left, there is a network diagram showing servers and computing devices needed to run these models, labeled with hardware such as TPU and GPU. On the right, the structure of BERT and GPT is compared in detail, including positional encoding, self-attention mechanisms, feed-forward networks, addition and normalization layers, and the computation of output probabilities. Although these models have different approaches to processing text, both are large neural network models based on deep learning and self-attention mechanisms. BERT: Bidirectional Encoder Representations from Transformers. GPU: graphics processing unit; LLM: large language model. TPU: tensor processing unit.

In health care, deploying large-scale medical models faces unique challenges due to data security and privacy concerns. Hospitals typically have CPUs for general computing, with limited access to GPUs. Medical LLMs, generally smaller than general-purpose LLMs, still require substantial investment in operational hardware [40,41]. For instance, a model with 13 billion parameters might cost under US $138,000 while larger models for entire hospitals may require advanced GPU solutions costing around RMB 10 million. Effective deployment demands careful consideration of model scale, computational resources, data security, and cost control (Figure 2).

## Advancing the Integration of LLMs in Health Care: The Imperative for Evidence-Based Research and Collaborative Evaluation

### Overview

In the contemporary health care landscape, the paradigm of evidence-based medicine is instrumental in shaping medical decision-making processes. This methodology integrates top-tier research evidence with clinical expertise and aligns it with patient values and expectations, thereby informing patient care decisions. Evidence-based medicine ensures that medical interventions are grounded in scientific evidence rather than solely relying on a physician’s experience or intuition, enhancing patient safety and the efficacy of treatments [42-45].

The integration of LLMs into the medical field introduces a significant challenge: the current scarcity of evidence-based medical research concerning the application of LLMs in health care settings [46]. Although LLMs have shown remarkable efficacy in various sectors, the unique context of medicine, with its direct implications for human life and health, necessitates a cautious approach to the introduction of untested technologies or methods into clinical practice [47]. Despite their robust data processing capabilities, LLMs present a potential risk for prediction errors in clinical environments. The medical domain, with its complex interplay of biology, physiology, and pathology, might be challenging for machine learning models to fully encapsulate, especially considering the intricacies and variability inherent in medical data [48]. Furthermore, the realm of medical decision-making often requires a high level of expertise and experience, aspects that may not be entirely replicable by LLMs. The consequences of medical decisions far surpass those in other sectors, where a misdiagnosis or incorrect treatment recommendation could directly jeopardize a patient’s life. Hence, it is imperative to back any new technological innovation, including LLMs, with solid scientific evidence before they are implemented in medical practice.

Currently, empirical studies examining the application of LLMs in the medical field are limited. This scarcity of research implies an inability to definitively assess the accuracy, reliability, and safety of LLMs within a health care context. Model reliability refers to the consistency and dependability of a model’s outputs across different datasets or under varying conditions. In medical applications, the reliability of LLMs is critical, as it directly affects the accuracy of diagnoses and treatment recommendations, where any inconsistency could have serious consequences for patient care. To comprehensively understand the potential benefits and risks associated with LLMs in medicine, a more robust body of clinical research is required. This research should encompass randomized controlled trials, observational studies, and extensive collaborative research, which are critical to evaluating the clinical utility of LLMs accurately [49].

To accelerate the empirical evaluation of LLMs in the medical field, fostering collaboration between medical institutions, research organizations, and technology companies is essential. This interdisciplinary collaboration ensures the comprehensiveness and quality of the research, facilitating the rapid advancement and application of LLM technologies. To enhance the transparency, trustworthiness, and ethical application of LLMs in health care, it is crucial to address the societal implications, particularly in terms of data privacy. Publicizing research findings and fostering interdisciplinary collaboration among doctors, researchers, and ethicists will be key to ensuring that LLMs are used responsibly and equitably. Furthermore, the integration of robust data privacy measures and adherence to ethical standards must be a priority to prevent potential misuse or unintended consequences that could undermine public trust. Such an approach ensures that LLMs’ application in the medical field is underpinned by scientific rigor, is safe, and genuinely benefits both patients and the health care system.

### Integrated Application of LLMs in Medical System

As we witness ongoing advancements in medical technology, the integration of LLMs with other tools and platforms within health care systems becomes increasingly crucial [50]. This fusion provides health care professionals with powerful tools to process, analyze, and effectively use vast amounts of health care data [23,51-54]. The integration of LLMs, such as ChatGPT, into medical systems has the potential to drive transformative progress in health care delivery. First, LLMs can potentially enhance diagnostic accuracy and clinical decision-making by analyzing comprehensive medical data to identify relevant information and suggest potential diagnoses based on presented symptoms [55-57]. Second, their proficiency in text processing and generation assists medical professionals in efficiently summarizing medical literature, facilitating research, and improving communication between health care providers and patients [58-61]. The rapid adoption of readily available LLMs, such as ChatGPT, within the medical community, signifies recognition of their potential to transform health care delivery [62-66].

However, the application of LLMs in clinical settings is not without challenges [67]. A primary concern is the generalizability of these models. Although LLMs have shown outstanding performance in numerous standard tasks, the complexity and diversity of the medical field suggest that these models may be susceptible to prediction errors in real clinical scenarios. Such errors can have serious implications, particularly when they influence critical health and life decisions. Additionally, the medical field encompasses a vast array of domain-specific knowledge that might exceed the training scope of LLMs, potentially leading to misunderstandings in complex medical scenarios.

**Figure 3:**
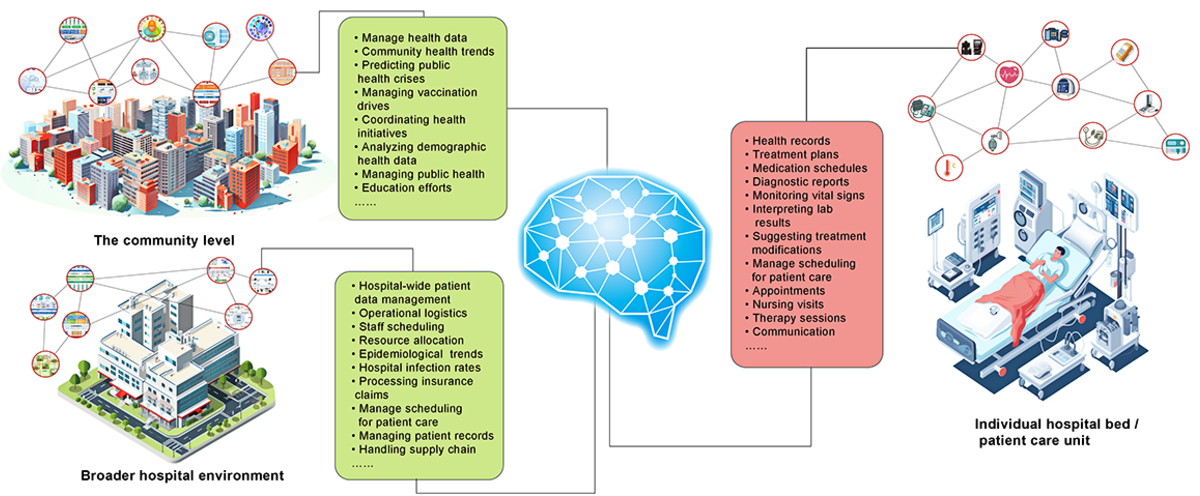
Integration of LLMs in health care systems across different scales. LLMs can assist in monitoring and analyzing patient health records, treatment plans, and laboratory results at the individual bed level while managing care schedules and facilitating doctor-patient communication. At the hospital level, LLM helps manage patient data, operational logistics, staff scheduling, and resource allocation, while analyzing epidemic trends and hospital infection rates. At the community level, LLM can be used to predict public health crises, manage vaccination campaigns, coordinate community health initiatives, and analyze population health data to improve health policy. LLM: large language model.

Despite these challenges, the potential benefits and impact of LLMs in health care are considerable. LLMs can notably enhance the efficiency of medical workflows by automating routine processes such as appointment scheduling, diagnosis, and report generation [68]. Their data-driven recommendations provide powerful decision support to doctors, assisting them in making more accurate and timely decisions. Current digital health workflows often burden physicians with extensive data entry, querying, and management tasks, leading to information overload and fatigue. LLMs can alleviate these burdens by automating these tasks, thereby saving valuable time for health care providers. Moreover, by analyzing and integrating patients’ medical data, LLMs can offer tailored diagnoses and treatment recommendations, improving the overall quality of health care delivery. LLMs also play a crucial role in enhancing doctor-patient interactions. Leveraging NLP technology, they can better comprehend patients’ needs and concerns, offering more personalized medical advice [69]. This not only boosts patient satisfaction but also enhances the overall effectiveness of medical services. The potential of LLMs to optimize digital health care workflows is undeniable. With further technological advancements and empirical research, LLMs are expected to play an increasingly significant role in the future of health care (Figure 3).

### Multimodal LLMs in Real-World Medical Scenarios

The advent of multimodal LLMs is bringing about a paradigm shift in the medical field by offering the capability to process and generate diverse data types such as text, images, sounds, and videos. This integration of multiple data types enables LLMs to provide more comprehensive and accurate predictions, thereby unlocking unprecedented potential [70-73]. To understand their role, it is essential to define what multimodal LLMs entail. Multimodal LLMs excel in processing, interpreting, and generating a wide array of data types, which significantly enhances their predictive capabilities. For instance, in the medical field, combining textual data from patient records with imaging data from magnetic resonance imaging (MRI), computed tomography scans, and x-rays allows these models to provide more nuanced and precise diagnoses. Additionally, integrating audio data from patient interviews or video data from medical procedures can further enrich the model’s understanding, leading to more accurate and personalized treatment recommendations. By leveraging the strengths of various data types, multimodal LLMs can offer a holistic view of a patient’s condition, which is often crucial for complex diagnoses and treatment planning.

The utility of LLMs is increasingly becoming a focal point in medical imaging [74-76]. For instance, when a patient undergoes an MRI or computed tomography scan, an LLM can swiftly analyze and integrate the image data with the patient’s textual medical records, thereby providing more comprehensive and detailed diagnostic insights. Additionally, LLMs have the capability to automatically identify and highlight crucial areas in medical images, thus providing clinicians with clear references that aid in identifying potential issues [77]. Moreover, LLMs can generate automated image reports, offering initial interpretations and treatment suggestions based on the analyzed image data, significantly boosting the efficiency and accuracy of medical diagnoses and treatments.

Multimodal LLMs are revolutionizing the field of telemedicine, transforming the dynamics of doctor-patient interactions [55,78]. For instance, LLMs have been successfully integrated into MRI analysis, where they can rapidly interpret imaging data and provide diagnostic recommendations. This has significantly reduced the time required for diagnosis and improved accuracy. However, the use of LLMs is not without its challenges. A notable example is Google BARD, which recently demonstrated racial bias in patient diagnosis, disproportionately affecting minority groups. This case highlights the dual-edged nature of LLMs in health care—they offer substantial benefits in efficiency and accuracy, yet they also pose significant risks if not properly validated and monitored for biases. Furthermore, the integration of LLMs with smart sensors and devices enables the continuous monitoring of patient’s physiological data, such as heart rate and blood pressure, facilitating early detection and intervention for any health anomalies, thus significantly bolstering patient health management.

**Figure 4:**
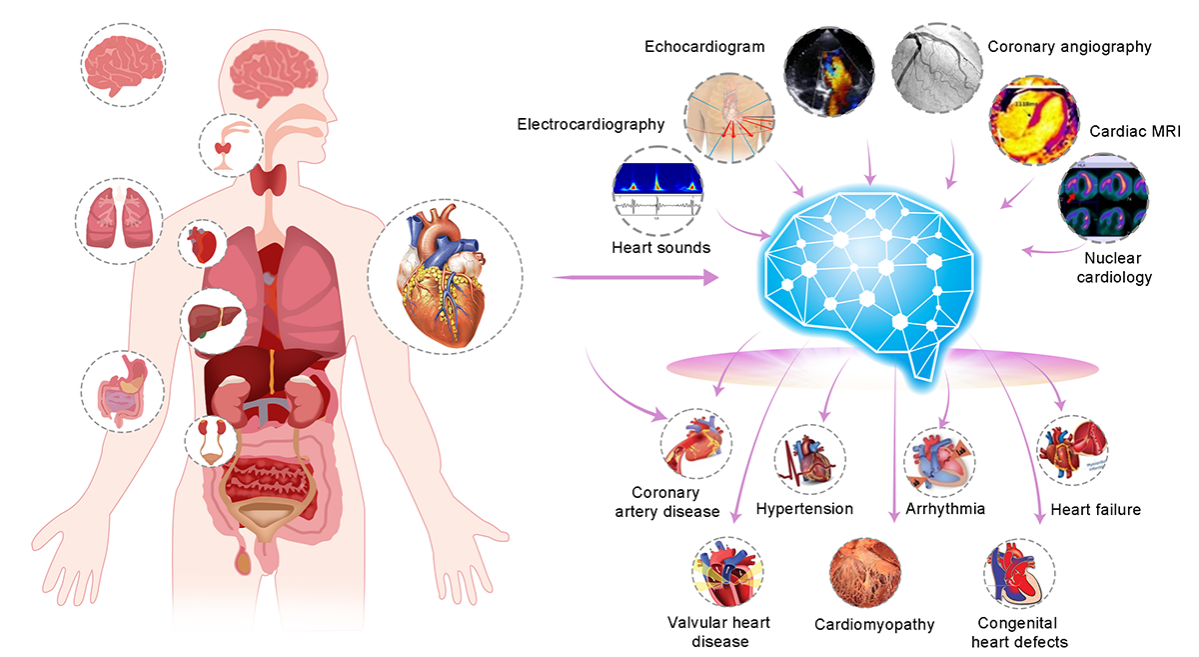
The importance of multimodal large language models in medical applications. The central heart represents the cardiac health status of the human body. The surrounding circular icons depict various cardiac conditions including coronary artery disease, hypertension, arrhythmia, heart failure, valvular heart disease, cardiomyopathy, and congenital heart defects. These conditions are detected and analyzed through different medical imaging and diagnostic technologies such as electrocardiography, heart sounds, echocardiogram, coronary angiography, cardiac MRI, and nuclear cardiology. The results from these diagnostics are processed by an AI system to determine the type and severity of cardiac disease, assisting physicians in formulating treatment plans. MRI: magnetic resonance imaging.

In summary, multimodal LLMs offer a novel and efficacious approach to diagnosis, treatment, and health care management. Their robust capabilities in data processing and integration allow medical professionals to deliver more precise and efficient services to patients. At the same time, these models enable patients to access medical advice and care with greater convenience. As these technologies continue to evolve and improve, their significance and impact in the medical field are expected to grow exponentially (Figure 4).

### The Key Role of LLMs in Medical Research

In the field of fundamental medical research, the capabilities of LLMs in AI are being increasingly recognized [79-82]. LLMs can swiftly retrieve and organize crucial information from vast biomedical literature, providing researchers with an efficient tool to access and synthesize the latest research findings on specific drugs, diseases, or genes [83]. In drug discovery, LLMs can predict the activity, toxicity, and pharmacokinetic properties of new compounds, facilitating early-stage drug screening [84]. These predictions not only save time but also facilitate the early-stage screening of potential drug molecules. LLMs can use existing literature and databases to predict the potential functions of newly discovered genes, a crucial aspect of genomic research, given the daily discovery and study of new genes. While protein structure prediction depends primarily on specialized models, such as AlphaFold, LLMs can enhance these models by supplying pertinent information from literature, thereby increasing prediction accuracy. In epidemiological research, LLMs can aid researchers in tracking and predicting disease spread by analyzing social media and other web-based text data, offering data support for public health decision-making. Finally, in bioinformatics applications, LLMs can assist researchers in predicting patterns, functional domains, and similarities to known biological sequences. Despite their extensive applications in biomedicine, LLMs cannot entirely replace laboratory experiments or in-depth biomedical expertise. Instead, they should be considered powerful supplementary tools, rather than replacements.

LLMs play a pivotal role in clinical research. They aid doctors and researchers by extracting essential information from medical records, and by organizing and categorizing data for easier analysis and application. For instance, they can expedite the selection of suitable patients for enrolment, thereby enhancing the design and implementation of clinical trials. In the role of a clinical research coordinator, these models assist with data entry, verification, and analysis, thereby accelerating the clinical research process. Through automated data processing and real-time analysis, LLMs can ensure data accuracy and completeness, while also reducing the workload of clinical research coordinators. This, in turn, speeds up the clinical research process and enhances research quality.

**Figure 5:**
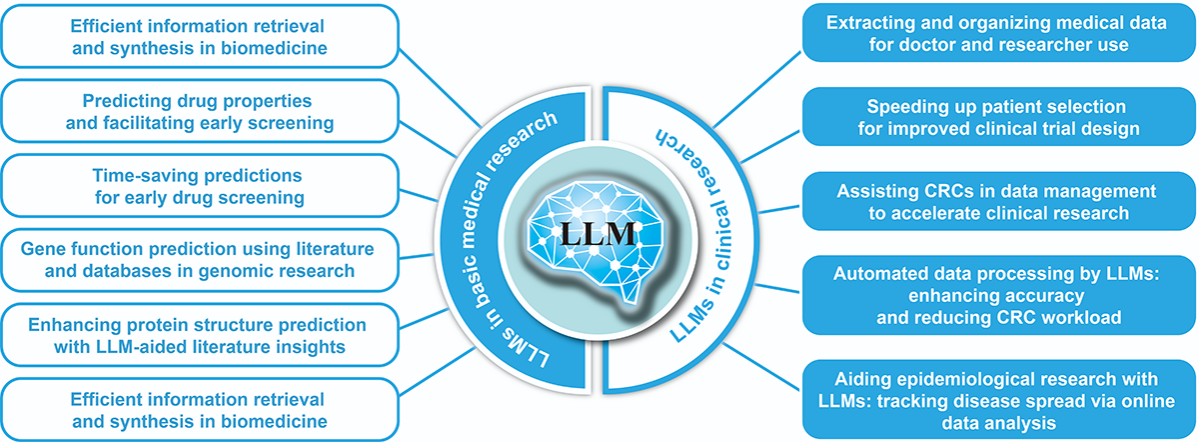
The crucial role of LLMs in medical science: bridging basic research and clinical trials. This illustration highlights the versatile roles of LLMs in medical research. LLMs analyze medical texts to uncover trends and inform research directions, facilitate hypothesis generation, and enhance clinical trial designs. They personalize medicine through data-driven treatment plans and use predictive modeling to inform clinical trial outcomes. LLMs also streamline research by integrating data and maintaining regulatory compliance. They assist in medical communication and education and evaluate the societal impact of clinical research. LLM: large language model.

Although LLMs have revolutionized biomedicine by simplifying literature searches, aiding drug discovery, annotating gene functions, and supporting epidemiological studies, they experience certain drawbacks. Their ability to swiftly parse large datasets and make predictions may be counterbalanced by potential limitations in real-world validation [85]. For example, while they can predict a drug molecule’s properties, the actual biological response may vary. Similarly, despite gene function predictions being well grounded, they may not fully encapsulate the breadth of gene interactions. Moreover, using LLMs to analyze epidemiological trends without correlating them to underlying data could misdirect public health interventions. Therefore, while LLMs are undeniably beneficial to biomedicine, it is essential to adopt a balanced approach, combining their computational prowess with rigorous experimental validation and expert review, to fully harness their potential without sacrificing scientific rigor (Figure 5).

### Great Challenges of LLMs in Medical Scenarios and Feasible Roadmap

The integration of technology in health care invariably brings a mix of anticipation and challenges, particularly given its direct impact on human life and health. As a leading exemplar of current AI technology, LLMs present a complex array of opportunities and challenges in the medical field, warranting thorough exploration and discussion [86-88].

Handling medical data, some of the most private and sensitive information about individuals, is a significant challenge for LLMs. As LLMs are increasingly integrated into health care, ethical considerations surrounding data privacy and societal impact must be prioritized during their development and deployment. The key lies not only in using this data to enhance medical efficiency but also in implementing robust data protection frameworks to prevent misuse, leakage, and unauthorized access. Furthermore, addressing these ethical challenges requires ongoing dialogue among technologists, health care professionals, policy makers, and the public to ensure that LLM deployment aligns with societal values and legal standards [80,89]. A potential technical solution involves anonymizing patient data, ensuring that neither processing nor transmission stages can be linked to specific individuals. Concurrently, medical organizations and technology providers must establish robust data management and access protocols, ensuring clear authorization and purpose for each data access.

Interpretive challenges loom large with LLMs in medicine. Medical decision-making is distinct from other fields due to its complexity and direct implications for patients’ lives and health. When LLMs provide diagnostic or treatment suggestions, it is vital that the rationale behind these recommendations is transparent and comprehensible [90-92]. This brings us to the concept of interpretability in machine learning, which refers to the ability to understand and explain how a model makes its decisions. In the context of health care, interpretability is a significant challenge because clinicians must trust and validate the outputs of LLMs, especially when these models influence critical medical decisions [93]. Developing mental models can aid LLMs in presenting their decision-making logic in a manner that is more accessible to human users. Leveraging deep learning and other machine learning technologies, LLMs can extract disease pathophysiological mechanisms from a vast corpus of medical literature and data, providing a scientific basis for their outputs. To further enhance interpretability, LLMs could use visual tools, like graphics and animations, to clarify the logic and evidence underpinning their decisions for both physicians and patients [94,95].

The issue of technical bias and the possibility of generating misleading information or “hallucinations” are inherent challenges in LLMs. In this context, hallucinations refer to instances where LLMs produce outputs that are factually incorrect or misleading, often because the model attempts to generate an answer despite lacking sufficient context or knowledge. These hallucinations can be especially problematic in medical scenarios, where inaccurate information can have severe consequences. The data sources for these models, often anonymized consultation data and digital materials, are not uniform and vary in quality, sometimes containing erroneous samples. Fine-tuning LLMs based on such data may lead to biased or skewed medical recommendations [96,97]. Addressing this requires rigorous data auditing and the establishment of continuous bias-correction mechanisms. To mitigate the risk of hallucinations, knowledge enhancement methods, such as integrating a knowledge retrieval library or search enhancement tools, can be beneficial. The LLM’s responses can be cross-referenced with retrieved data to filter out inconsistencies with reality. Another approach involves reinforcement learning based on human feedback, where high-quality feedback is provided to fine-tune and correct model outputs in collaboration with medical experts [98,99].

The potential of AI to create “information cocoons” through personalized content, potentially reinforcing biases, is another critical aspect that needs to be addressed, especially in the medical domain [100]. AI technologies, including LLMs, in medicine require stringent scrutiny and continuous evaluation to align with the field’s unique characteristics and ethical standards. Ensuring privacy protection, eliminating biases and discrimination, and establishing clear accountability are essential. The use of LLMs should be guided by respect for life, aiming to enhance patient well-being and treatment outcomes, without compromising individual interests. A continuous monitoring and evaluation system is crucial for assessing the effectiveness of LLMs and managing potential risks. Regulations should be regularly updated to keep pace with AI advancements, ensuring medical safety and patient rights. By prioritizing safety, fairness, and effectiveness, we can fully leverage LLMs and other AI technologies to facilitate a transformative revolution in medicine, while upholding human values and rights.

In the era of information and intelligence within the medical field, the application of LLMs harbors immense potential [101]. However, the accompanying challenges are equally noteworthy and merit careful consideration. The ongoing discourse should emphasize not only the deeper integration of LLMs into medical practice but also their alignment with both the professional needs of health care providers and the experiential needs of patients [102,103].

Incorporating the theory of mind into LLMs can significantly enhance their utility in the medical field. This concept, which involves understanding others’ thoughts, feelings, and intentions, is crucial for fostering trust and empathy within health care interactions. Medicine is not solely a science; it is also an art, deeply influenced by each patient’s unique emotional, value-based, and experiential landscape. An AI system endowed with the capability to appreciate and respond to these individual differences can offer more personalized and compassionate medical advice [104,105]. By using the theory of mind, LLMs can gain deeper insights into patients’ inherent needs and respond with more attentive and empathetic advice [106-108]. When LLMs can emulate the thoughts and feelings of both doctors and patients, their outputs transcend mere data; they become imbued with empathy and human care, enhancing the patient’s treatment experience and fostering stronger trust and communication between doctors and patients. For example, in interactions with terminal patients, LLMs could suggest more compassionate communication strategies, aiding both doctors and patients in navigating these sensitive and complex situations.

LLMs can be synergistically combined with other advanced technologies, such as virtual reality and augmented reality, to transform medical consultations into more immersive and informative experiences. This integration can provide patients with a deeper understanding of their health conditions, empowering them to make more informed decisions regarding their treatment. The evolution of LLMs is also contingent upon the development of efficient and precise algorithms capable of adeptly handling complex medical data, which is essential for accurate and timely medical decision-making. As technology progresses, the use of LLMs in the medical field is expected to become increasingly intelligent, efficient, and personalized, thereby enhancing not only the quality of medical services but also the overall patient experience and driving the evolution and transformation of the health care industry.

In our pursuit of technological progress, we must adhere to a fundamental principle: ensuring that technology is accessible to all. This is particularly pertinent in the context of LLM adoption, where it is crucial not to overlook those who may be marginalized by the technology gap [109,110]. Whether addressing the needs of rural farmers or urban older adults, every individual should have the opportunity to benefit from LLMs. This broad adoption must span various geographical regions and encompass diverse languages and cultural contexts, catering to users speaking English, Chinese, or local dialects [111,112]. Achieving this objective is not solely a technological challenge but also a social imperative. We must ensure that the design and application of LLMs overcome language and cultural barriers, truly reaching and benefiting a diverse global populace. Additionally, addressing technology accessibility issues is vital. For individuals in technologically underserved areas or older adults unfamiliar with new technologies, simpler access methods and more user-friendly interfaces are needed to facilitate effortless use of LLMs.

While the potential of LLMs in health care is significant, realizing this potential requires ongoing research, innovation, and dedication. Continuous efforts are necessary to refine LLM technology continually and ensure its broad adoption across all sectors of society. We firmly believe that with sustained commitment, LLMs will catalyze transformative changes in health care, benefiting society at large. By championing technological inclusivity, we can not only enhance the quality and efficiency of medical services but also promote overall societal health and well-being.

### Economic Considerations in the Deployment of LLMs in Health Care

LLMs require significant computational resources for training and maintenance, which translates to substantial financial costs. In the medical domain, these costs can be particularly prohibitive due to the need for specialized data, high levels of accuracy, and continuous updates to ensure model relevance and safety.

Training a state-of-the-art LLM, such as GPT-3, requires access to extensive hardware infrastructure, including thousands of GPUs or TPUs, large amounts of RAM, and high-speed data storage solutions [113,114]. According to estimates, the training cost of models, like GPT-3, can reach up to US $1.4 million, with operational costs amounting to several hundred thousand dollars per day when deployed at scale. In a medical context, where accuracy and reliability are paramount, these costs are even higher due to the additional requirements for data security, privacy, and compliance with health care regulations.

Several studies have documented the economic challenges associated with deploying LLMs in health care [115,116]. For instance, the cost of implementing LLMs in hospital settings, including the necessary infrastructure upgrades, staff training, and ongoing maintenance, has been reported to be a major barrier to widespread adoption. Moreover, the need for regular updates to the models, which involves retraining them with new medical data, adds to the operational expenses [1].

As technology advances, it is expected that the costs associated with LLMs will decrease, making them more accessible to a broader range of health care providers. The development of more energy-efficient hardware, combined with advances in machine learning techniques, is likely to contribute to this trend. However, until these cost reductions are realized, careful planning and resource allocation will be essential for any institution looking to implement LLMs in their health care practice.

### Conclusions

The era of digitalization and informatization underscores the transformative potential of LLMs in medicine. The evolution of this technology signifies a paradigm shift in medical services, offering unique opportunities and challenges to the medical community. LLMs, with their advanced NLP capabilities, have a wide range of applications including emergency triage, older people care, and the enhancement of digital medical workflows. As the diversity of medical data expands, LLMs’ ability to process multimodal data will play a crucial role in enabling more precise, personalized medical diagnoses and treatments.

Despite the promising trajectory of LLMs in the medical field, ensuring their safety and effectiveness in clinical practice remains a critical challenge. Currently, the regulation of LLMs in health care is still in its early stages, with several frameworks being developed to address the unique risks and challenges they pose. Regulatory bodies, such as the US Food and Drug Administration, European Medicines Agency, and China’s National Medical Products Administration, have begun to formulate guidelines that apply to AI-driven medical devices including LLMs. These guidelines typically focus on the validation of the models through rigorous clinical trials, ensuring that they meet specific safety, efficacy, and ethical standards before they can be deployed in clinical settings. However, the growth potential of LLMs in the medical arena is significant. They can enhance patient experiences through the integration of virtual reality and augmented reality, offer comprehensive medical advice through multimodal research, and humanize doctor-patient interactions using the theory of mind. With ongoing advancements in algorithms and computational power, we anticipate considerable improvements in LLMs’ processing speed and accuracy.

However, the path to technological advancement is not always linear. To ensure the benefits of LLMs are accessible to all, it is imperative to promote equitable development and address the digital divide, particularly for economically and technologically disadvantaged regions and groups. This goal requires the collective efforts of health care professionals, computer science experts, government regulatory bodies, patients, and their families. Such a collaborative approach will ensure that the application of LLM technology in the medical field genuinely contributes to the betterment of humanity, significantly enhancing health and well-being.

## Abbreviations

AI: artificial intelligence
BERT: Bidirectional Encoder Representations from Transformers
GPU: graphics processing unit
LLM: large language model
MRI: magnetic resonance imaging
NLP: natural language processing
TPU: tensor processing unit
